# Ipsilateral Aorto-Iliac Calcification is Not Directly Associated With eGFR After Kidney Transplantation: A Prospective Cohort Study Analyzed Using a Linear Mixed Model

**DOI:** 10.3389/ti.2023.10647

**Published:** 2023-01-20

**Authors:** Elsaline Rijkse, Joke I. Roodnat, Sara J. Baart, Diederik C. Bijdevaate, Marcel L. Dijkshoorn, Hendrikus J. A. N. Kimenai, Jacqueline van de Wetering, Jan N. M. IJzermans, Robert C. Minnee

**Affiliations:** ^1^ Department of Surgery, Division of HPB and Transplant Surgery, Erasmus MC Transplant Institute, Erasmus MC University Medical Center, Rotterdam, Netherlands; ^2^ Department of Internal Medicine, Division of Nephrology and Transplantation, Erasmus MC Transplant Institute, Erasmus MC University Medical Center, Rotterdam, Netherlands; ^3^ Department of Biostatistics, Erasmus Medical Center, Rotterdam, Netherlands; ^4^ Department of Radiology, Erasmus MC University Medical Center, Rotterdam, Netherlands

**Keywords:** kidney transplantation, kidney transplant recipients, peripheral artery disease, kidney transplant outcomes, chronic kidney disease

## Abstract

Aorto-iliac calcification (AIC) is a well-studied risk factor for post-transplant cardiovascular events and mortality. Its effect on graft function remains unknown. The primary aim of this prospective cohort study was to assess the association between AIC and estimated glomerular filtration rate (eGFR) in the first year post-transplant. Eligibility criteria were: ≥50 years of age or ≥30 years with at least one risk factor for vascular disease. A non-contrast-enhanced CT-scan was performed with quantification of AIC using the modified Agatston score. The association between AIC and eGFR was investigated with a linear mixed model adjusted for predefined variables. One-hundred-and-forty patients were included with a median of 31 (interquartile range 26–39) eGFR measurements per patient. No direct association between AIC and eGFR was found. We observed a significant interaction between follow-up time and ipsilateral AIC, indicating that patients with higher AIC scores had lower eGFR trajectory over time starting 100 days after transplant (*p* = 0.014). To conclude, severe AIC is not directly associated with lower post-transplant eGFR. The significant interaction indicates that patients with more severe AIC have a lower eGFR trajectory after 100 days in the first year post-transplant.

## Graphical Abstract

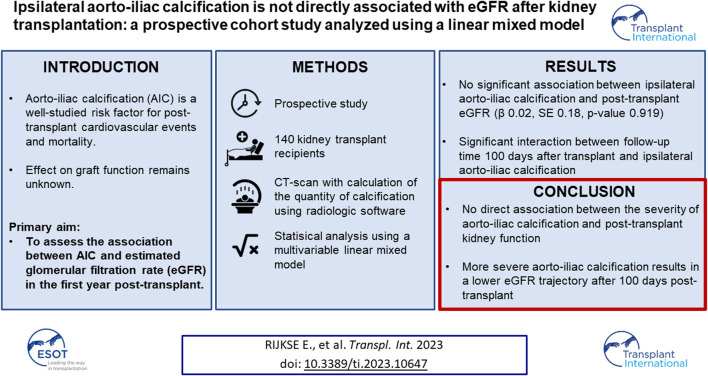

## Introduction

Vascular disease is prevalent in patients with end-stage renal disease (ESRD) due to the high incidence of traditional risk factors and chronic kidney disease (CKD) related factors, such as CKD-related mineral and bone disorder (1, 2). Vascular calcification was thought to occur primarily during ESRD, but recently it has been found that its development begins in earlier stages of CKD (1). The pathophysiological mechanism consists of both arteriosclerosis and atherosclerosis, of which arteriosclerosis is characterized by vascular stiffening and atherosclerosis by intimal wall thickening (3). Even though both subtypes of vascular disease are highly prevalent in CKD patients, arteriosclerosis is the most strongly linked to CKD (4). Vascular disease manifests clinically as coronary artery disease, cerebrovascular disease and peripheral arterial disease. As a result of this increased prevalence, mortality from cardiovascular disease is 10–20 times higher in patients with ESRD compared to the general population (5).

Vascular disease can also occur in the aorto-iliac arteries, resulting in an increased need for intra-operative vascular reconstructions (6). As a consequence, aorto-iliac vascular disease is the main reason for decline for kidney transplantation (7). Given that AIC is a manifestation of generalized vascular disease, it is not surprising that several studies have shown that AIC is associated with inferior survival and an increased risk of post-transplant cardiovascular events (8, 9). However, little is known about the association between ipsilateral AIC and graft function. This information is important, as a large retrospective cohort study found that 25% of all kidney transplant candidates presented with any degree of AIC (10).

Current studies that investigated the relationship between AIC and graft function have several limitations, from which the most important ones are a retrospective design, a subjective quantification method of aorto-iliac vascular disease which limits generalizability and the use of statistical methods that do not account for drop-out (10-16). To address these issues, we performed a prospective cohort study in which all patients underwent non-contrast-enhanced CT-scan for objective, quantitative assessment of AIC using an adaptation of the Agatston score. This score is widely used to quantify coronary artery calcification and has excellent inter-observer and inter-scanner agreement (17, 18). The primary aim of our study was to investigate the association of ipsilateral AIC with post-transplant estimated glomerular filtration rate (eGFR) trajectory in the first year post-transplant using a linear mixed model.

## Materials and Methods

### Study Design and Eligibility Criteria

This prospective, single-center study was carried out in Erasmus Medical Center, the Netherlands, between 10 January 2019 and 13 August 2020. Power calculation for the study sample size can be found in the Supplementary Material. Patients who met the eligibility criteria were asked to participate upon admission for transplant. All patients 50 years or older were eligible for inclusion. Patients from 30 years of age or older were eligible if they had at least one of the following risk factors for vascular disease: diabetes mellitus, 1 year or longer dialysis duration, smoking history of at least 10 pack years or a history with peripheral arterial disease, ischemic heart disease or a cerebrovascular accident (19). In addition, South-East Asian ethnicity was considered a risk factor as previous studies found that these patients were at increased risk of cardiovascular disease after adjustment for confounders (20). Combined liver-kidney transplant recipients, HLA incompatible recipients and dual transplant recipients were excluded from the study. The study was performed according to the Declaration of Helsinki and received approval from the local Medical Ethical Committee (MEC 2018-1401). The study was prospectively registered in the Netherlands Trial Register (NTR7641).

### Study Procedure

**FIGURE 1:**
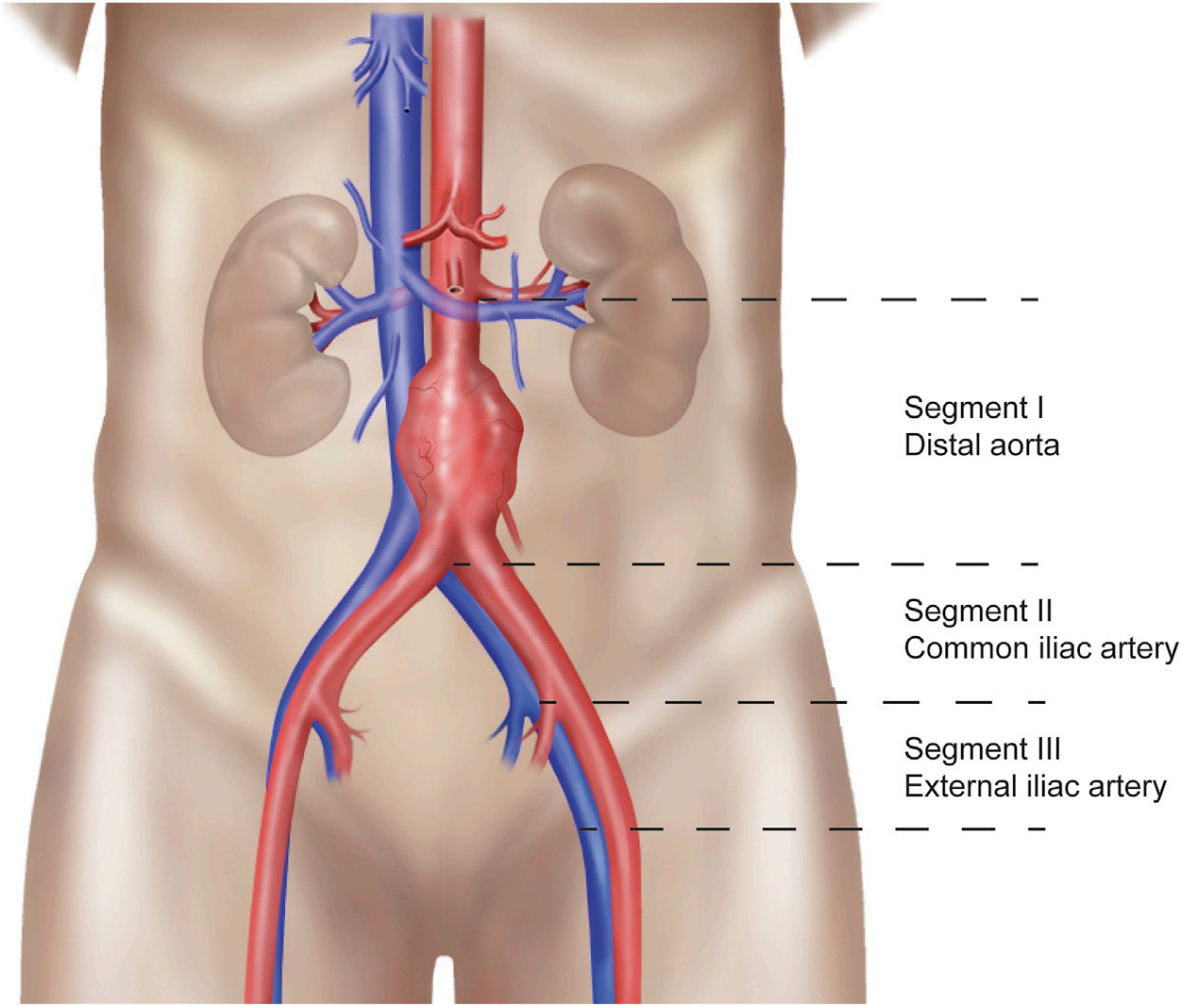
Explanation of the anatomical segments.

Patients who gave written informed consent underwent a non-contrast-enhanced abdominal CT-scan upon admission for transplant. All scanners used were modern ≥128-multislice CT systems (Siemens Healthineers). A scanning protocol was developed specifically for the study to ensure that there would be no differences in scan parameters that could affect measurement of the calcification score. A low-dose CT-scan (at fixed 120 kVp) was performed and reconstructed to 3.0 mm slice thickness and 1.5 mm increment using a dedicated quantitative calcium scoring kernel (B35f or Qr36) without iterative reconstructions. Consequently, the scan was analyzed in Intellispace Portal (Philips) with the HeartBeat-CS application. This application is designed to calculate the Agatston score, which is a continuous quantification score for coronary calcification with a high specificity for the absence of coronary artery disease (21, 22). The calculation is based on the weighted density score given to the highest attenuation value multiplied by the area of the calcification speck. A CT attenuation threshold of 130 Hounsfield units (HU) is used for the detection of calcification, with only contiguous voxels totaling ≥1 mm^2^ in area counted as lesions to reduce the influence of image noise(21). For calculation of the AIC score as an adapted version of the Agatston score, the aorto-iliac trajectory was divided into anatomical segments, as explained in Figure 1. These anatomical segments included the infrarenal aorta until the iliac bifurcation (segment I), the right and left common iliac artery until the internal iliac artery branch (segment II), and the right and left external iliac artery (starting from the internal iliac artery branch until Poupart’s ligament) (segment III). The total AIC score was calculated as the sum of these separate calcification scores. The ipsilateral AIC score consisted of the sum of the aorta and ipsilateral common iliac artery, depending on the implantation side. The external iliac artery calcification score was not included in this score for 2 reasons. Firstly, the external iliac artery is not entirely in the inflow trajectory of the donor kidney, depending on whether the anastomosis is made with the proximal or distal part. Secondly, the transition from external iliac artery to common femoral artery was often unclear because of the use of a low-dose CT-scan which could result in misclassification.

### Primary and Secondary Outcomes

The primary outcome of the study was eGFR in the first year post-transplant. All measurements of eGFR from day 1 until 400 days were used, as not all patients had their 1-year eGFR measurement exactly at 365 days post-transplant. eGFR was calculated using the Chronic Kidney Disease Epidemiology Collaboration (CKD-EPI) formula, which is standard in our hospital as recommended by the kidney disease improving global outcomes (KDIGO) guidelines (23, 24). Secondary outcomes were the incidence of delayed graft function (DGF), primary non-function (PNF), the presence and number of biopsy-proven acute rejection episodes in the first year post-transplant, uncensored and death-censored graft survival, and the need for a peri-operative or pre-operative vascular intervention.

### Standard Transplantation Procedure

All donor kidneys were transplanted into the right or left iliac fossa by using the standard Gibson incision, depending on surgeon preference. Firstly, the anastomosis of the renal vein was performed end-to-side with the external iliac vein. Consequently, the arterial anastomosis was performed end-to-side with the external iliac artery using prolene 5.0 or 6.0. In the case of severe aorto-iliac calcification without a soft spot to implant the kidney, an endarterectomy could be performed with patch angioplasty prior to the arterial anastomosis. For the ureter, an extra-vesical anastomosis was performed as described by Lich-Gregoir. The ureter anastomosis was protected with an external splint or double J stent, depending on randomization arm of an ongoing randomized controlled trial investigating urologic complications. Standard immunosuppression regime of transplant recipients consisted of induction with basiliximab followed by triple-therapy with tacrolimus, mycofenolate mofetil and prednisone. Prednisone was gradually tapered and stopped after 4 months.

### Statistical Analysis

Continuous variables were presented as median and interquartile range (IQR) for non-normally distributed variables and mean with standard deviation for normally distributed variables. Categorical variables were summarized as number and percentage. Baseline characteristics were compared between patients with a median or lower ipsilateral AIC score and patients with an above median AIC score. Categorical baseline characteristics were compared with chi-square tests or Fisher’s exact test. Continuous baseline characteristics were compared using Mann-Whitney U test. Correlations between arterial segments were calculated using Spearman’s correlation coefficient. The association between the ipsilateral AIC score as a continuous variable and eGFR trajectory was analyzed using a linear mixed model with random intercepts and random slopes. An unstructured covariance matrix was used because of unbalanced outcome data. We used a predefined model to correct for donor, recipient and transplant-related confounders. We included the following variables in our fixed effects: follow-up time, ipsilateral AIC score, recipient age, recipient sex, recipient diabetes, recipient smoking, a previous kidney transplant, total dialysis duration (including hemodialysis and peritoneal dialysis), coronary artery disease, peripheral arterial disease, donor type, donor age, donor diabetes, donor last creatinine, pre-emptive transplantation, cold preservation technique (static cold storage or hypothermic machine perfusion), total human leukocyte antigen (HLA) mismatch, virtual panel reactive antibodies (vPRA), cold ischemic time, postoperative dialysis and 1 or more rejection episodes in the first year after transplant. The univariable analysis can be found in the Supplementary Table S1. In case of non-linearity for continuous variables, a natural cubic spline with 3 degrees of freedom was used (25). Knots for splines were selected based on the observed non-linear trajectories. Multicollinearity was investigated by calculating the generalized variance inflation factor (GVIF), where a value below 5 indicates no multicollinearity. The following clinically plausible interactions were tested with likelihood ratio tests: time and ipsilateral calcification score, time and donor type and time and delayed graft function. The main analysis included all eGFR measurements of the recipients, independent of graft failure. We performed one sensitivity analysis where we imputed an eGFR of 10 mL/min/1.73 m^2^ after graft failure occurred if the measured eGFR was above 10 mL/min/1.73 m^2^. Secondary outcomes were analyzed using unadjusted analyses due to a lack of statistical power for these outcomes. Differences in survival were calculated with the log-rank test. Median follow-up time was calculated with the reversed Kaplan-Meier method. R statistical software version 4.0.4. was used for data analysis (packages “nlme,” “lme4,” “splines2,” “survival”).

## Results

### Selection of the Cohort

**FIGURE 2:**
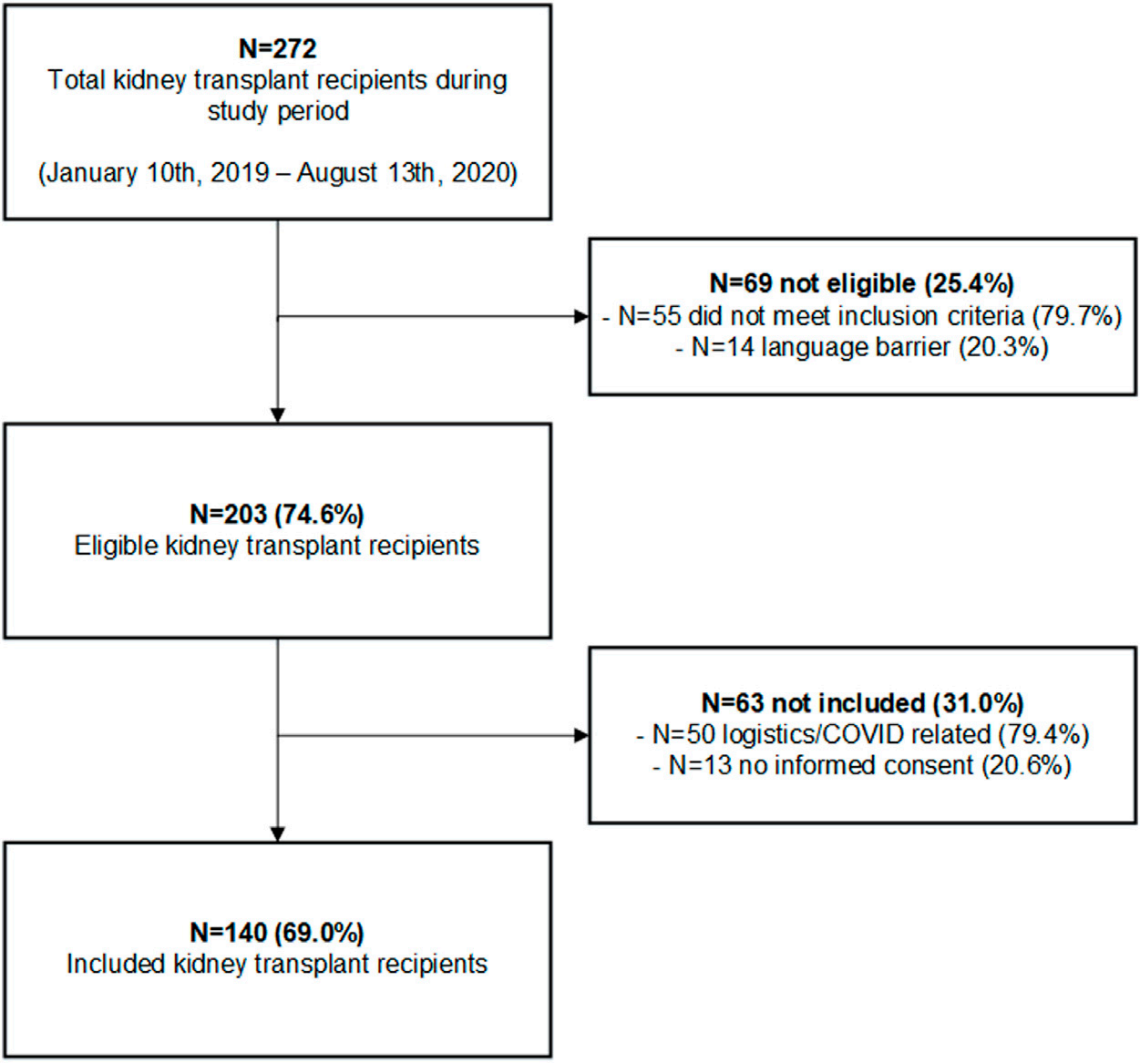
Study flowchart (*n* = 140).

A total of 140 kidney transplant recipients were included in the study and received a non-contrast-enhanced CT-scan. The inclusion flowchart is shown in Figure 2. 272 patients received a kidney transplant during the inclusion period of which 69 did not meet the inclusion criteria. Two-hundred-and-three patients were eligible for inclusion with a consent rate of 69.0%. The consent rate was lower than expected due to logistical reasons and a mandatory study stop due to the COVID-19 pandemic. Median follow-up time in the cohort was 622 days (IQR 416-757).

### Overall Calcification Scores

**FIGURE 3:**
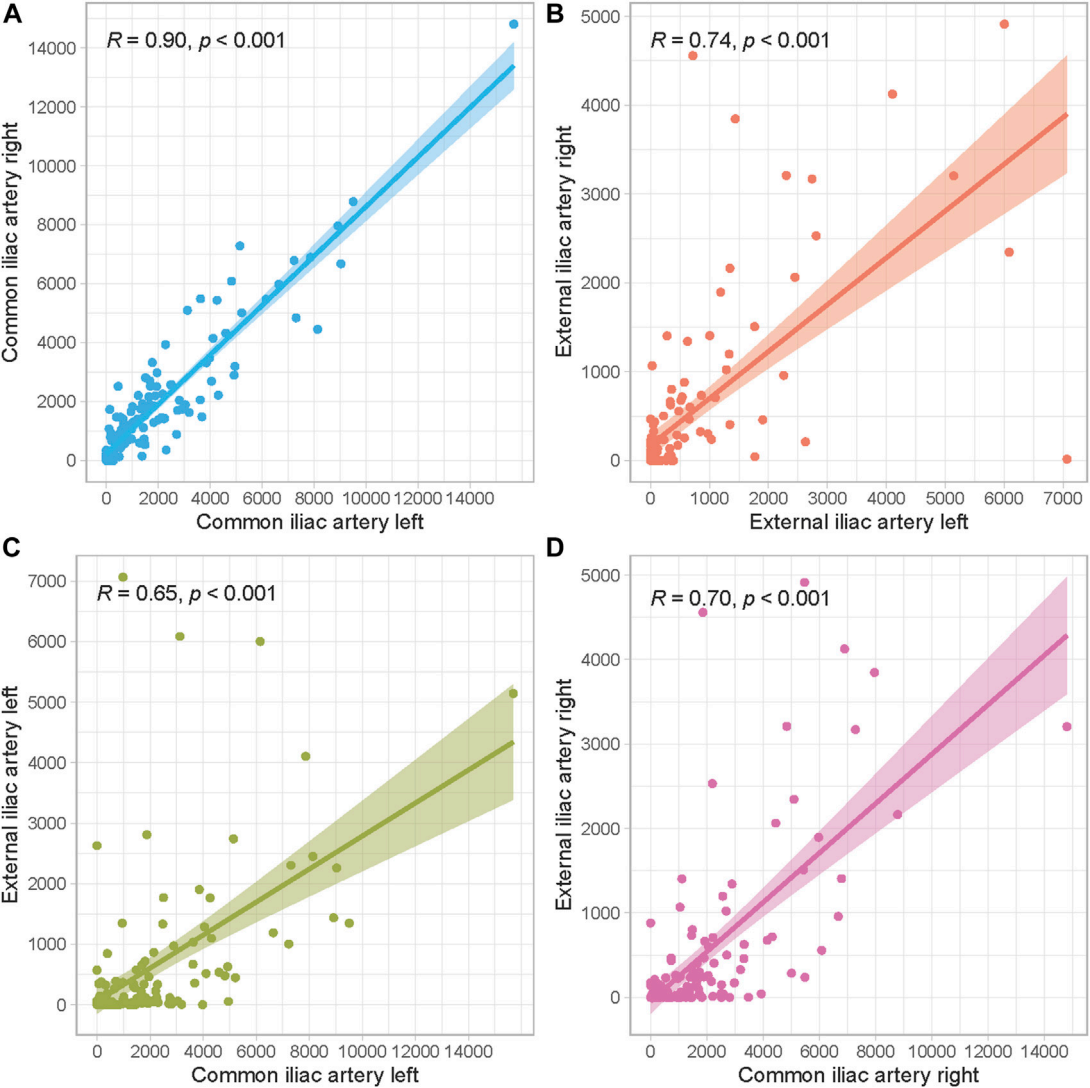
Scatter plots to assess correlations between arterial segments. **(A)** Left and right common iliac artery, **(B)** Left and right external iliac artery, **(C)** Left common and external iliac artery, **(D)** Right common and external iliac artery.

Median AIC scores per segment are illustrated in Table 1. The right and left external iliac artery had the lowest calcification scores with a median of 48 (IQR 0–412) and 52 (0–466), respectively. Scatter plots to identify correlations between calcification scores of separate arterial segments are presented in Figure 3. All AIC scores of arterial segments were highly correlated, but the highest correlation was found between the right and left common iliac artery (Spearman’s *ρ* = 0.90, *p* < 0.001). The lowest correlation coefficients were found between the right external and common iliac artery (Spearman’s *ρ* = 0.70, *p* < 0.001) and left external and common iliac artery (Spearman’s *ρ* = 0.65, *p* < 0.001).

**TABLE 1:** Calcification scores per segment in the whole cohort (*n* = 140).

Arterial segment		Left	Right
Aorta, median (IQR)	2,730 (754–7,135)		
Common iliac artery, median (IQR)		930 (154–2,288)	1,065 (152–2,211)
External iliac artery, median (IQR)		52 (0–466)	48 (0–412)
Total ipsilateral, median (IQR)	4,241 (1,144–10,221)		
Total, median (IQR)	5,451 (1,755–13,252)		

IQR, interquartile range.

### Baseline Characteristics

Baseline characteristics of the cohort are displayed in Table 2. To demonstrate differences between patients with a low or high ipsilateral AIC score, baseline characteristics were compared according to the median AIC score. Recipient age upon transplantation was significantly higher in recipients with a high ipsilateral calcification score (median age 66.5 (IQR 61.8–72.5) compared to 63.2 (IQR 54.7–68.7), *p* = 0.005). Patients with a high ipsilateral AIC score were more often current or former smokers (overall *p* = 0.007). Furthermore, patients with a high AIC score were more often presenting with a history of coronary artery disease (*p* < 0.001) or cerebrovascular disease (*p* = 0.039). Other baseline characteristics were not statistically different.

**TABLE 2:** Baseline characteristics from the cohort, stratified according to the median ipsilateral AIC score.

	Low AIC score (≤4,241)	High AIC score (>4,241)	*p*-value
	N = 70	N = 70	
Recipient-related			
Age (years), median (IQR)	63.2 (54.7–68.7)	66.5 (61.8–72.5)	0.005
BMI (kg/m^2^), median (IQR)	27.3 (23.9–30.8)	27.3 (24.5–32.5)	0.363
Sex			0.224
Male, n (%)	39 (55.7)	47 (67.1)	
Female, n (%)	31 (44.3)	23 (32.9)	
Smoking			0.007
Never, n (%)	35 (50.0)	17 (24.3)	
Currently, n (%)	9 (12.9)	15 (21.4)	
Former, n (%)	26 (37.1)	38 (54.3)	
Total dialysis duration (months), median (IQR)	7 (0–24.8)	11 (0–28.8)	0.247
Diabetes, n (%)	27 (38.6)	30 (42.9)	0.731
Hypertension, n (%)	58 (82.9)	62 (88.6)	0.469
Coronary artery disease			<0.001^a^
None, n (%)	67 (95.7)	39 (55.7)	
Single vessel, n (%)	0 (0.0)	14 (20.0)	
Double vessel, n (%)	1 (1.4)	7 (10.0)	
Triple vessel, n (%)	2 (2.9)	10 (14.3)	
Cerebrovascular disease, n (%)			<0.039^a^
None, n (%)	65 (92.9)	56 (80.0)	
TIA, n (%)	1 (1.4)	8 (11.4)	
CVA, n (%)	4 (5.7)	6 (8.6)	
Previous transplant, n (%)	12 (17.1)	11 (15.7)	1.000
COPD			0.245
No, n (%)	69 (98.6)	66 (94.3)	
GOLD I, n (%)	1 (1.4)	1 (1.4)	
GOLD II, n (%)	0 (0.0)	3 (4.3)	
Peripheral arterial disease, n (%)	1 (1.4)	7 (10.0)	0.063^a^
Donor-related			
Donor type			0.420
Living, n (%)	39 (55.7)	35 (50.0)	
DCD, n (%)	19 (27.1)	26 (37.1)	
DBD, n (%)	12 (17.1)	9 (12.9)	
Donor WIT (minutes), median (IQR)	3 (2-9)	4 (3-14)	0.129
Age (years), median (IQR)	56.0 (48.3–64.8)	63.0 (48.0–71.8)	0.079
BMI (kg/m^2^), median (IQR)	25.0 (23.3–29.3)	26.0 (24.0–28.2)	0.695
Diabetes, n (%)	2 (2.9)	2 (2.9)	1.000
Hypertension, n (%)	14 (20.0)	22 (31.4)	0.176
Last creatinine (µmol/L), median (IQR)	69.0 (59.3–86.5)	72.0 (62.3–81.0)	0.793
Transplant-related			
Pre-emptive transplant, n (%)	37 (52.9)	49 (70.0)	0.056
HLA mismatch, median (IQR)	3 (2-5)	4 (3-5)	0.269
vPRA, median (IQR)	0 (0–24)	0 (0–0)	0.362
AB0i transplant, n (%)	2 (2.9)	1 (1.4)	1.000
Ureteral stent			0.729
Single J, n (%)	41 (58.6)	44 (62.9)	
Double J, n (%)	29 (41.4)	26 (37.1)	
Anastomosis time (minutes), median (IQR)	21.0 (16.3–25.8)	23.0 (16.0–28.0)	0.453
Cold ischemic time (minutes), median (IQR)	142.5 (114.3–656.8)	350.0 (130.3–656.8)	0.235

^a^ Fisher’s exact test.

AB0i, AB0 incompatible; BMI, body mass index; COPD, chronic obstructive pulmonary disease; CVA, cerebrovascular disease; DBD, donation after brain death; DCD, donation after circulatory death; GOLD, global initiative for obstructive lung disease; IQR, interquartile range; TIA, transient ischemic attack; vPRA, virtual panel reactive antibodies; WIT, warm ischemic time.

### Longitudinal Trajectory of eGFR

**FIGURE 4:**
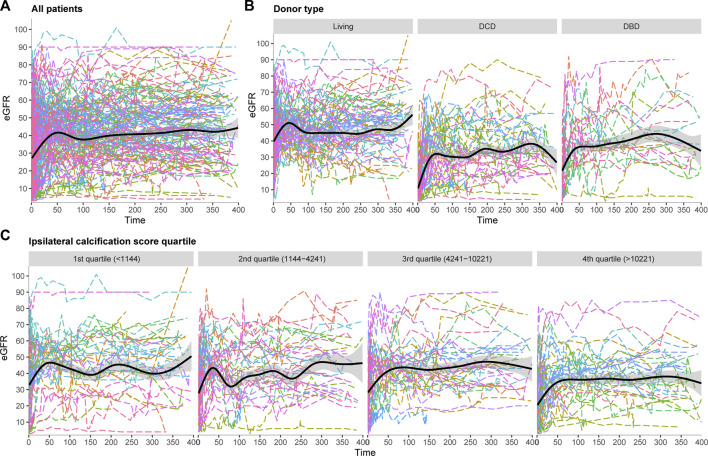
Spaghetti plots for eGFR trajectory in the first year after transplant, **(A)** All patients, **(B)** Stratified according to donor type, **(C)** Stratified according to ipsilateral calcification score.

Patients had a median of 31 (IQR 26–39) eGFR measurements available in the first year after transplant. Figure 4 shows the spaghetti plot for eGFR trajectory, stratified according to donor type (Figure 4B) or AIC score quartile (Figure 4C). All AIC quartiles showed a similar pattern with a steep increase in the first period followed by a small decrease and stabilization phase. eGFR trajectories during the stabilization phase were visually different with a lower eGFR trajectory in the highest calcification quartile.

### Linear Mixed Model for eGFR Trajectory After Transplant

**FIGURE 5:**
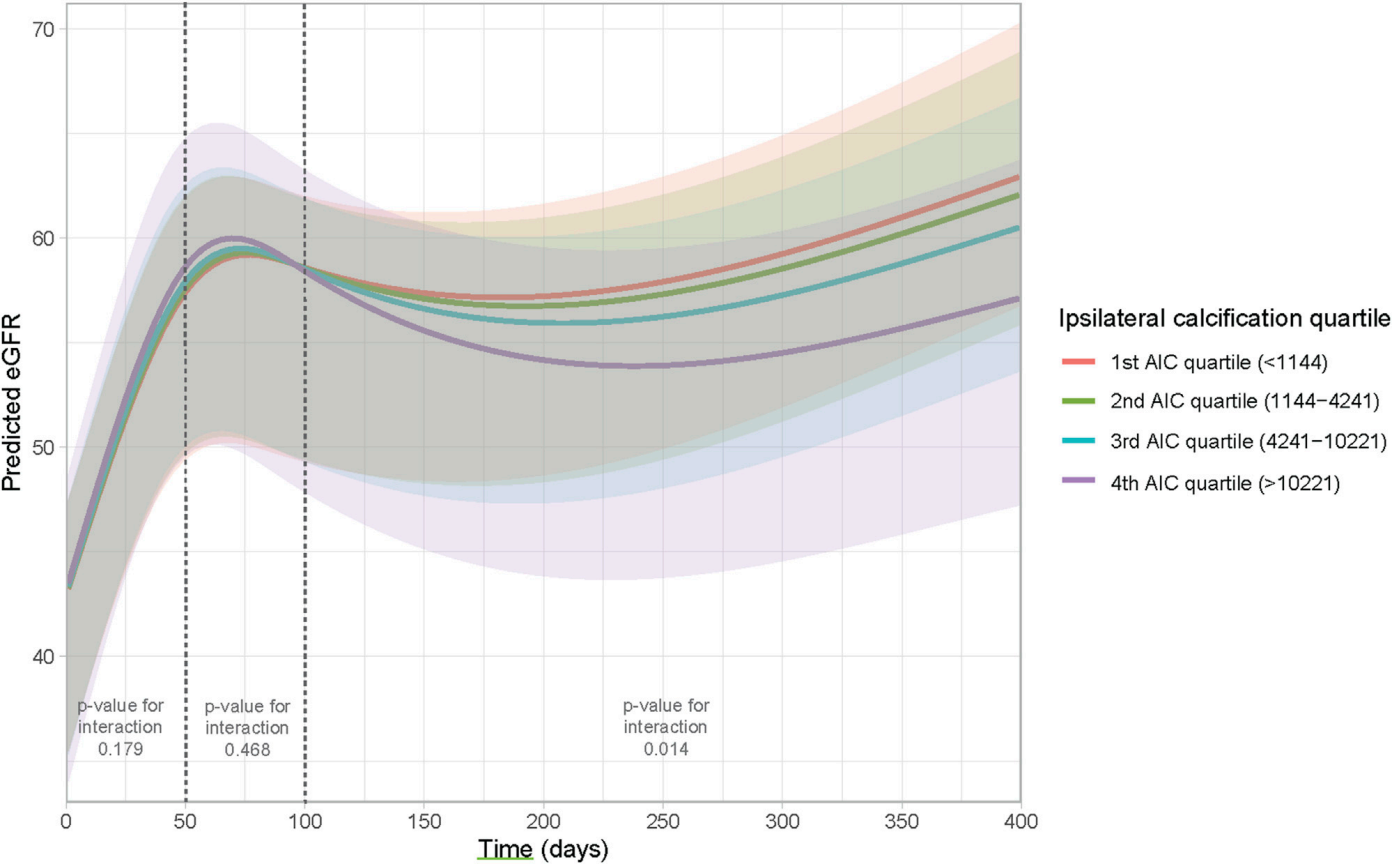
Effect plot to visualize the interaction between time and ipsilateral AIC score. The Y-axis represents the predicted eGFR based on the model, the X-axis represents time.

To assess whether this visual difference in eGFR trajectory during the first year post-transplant could also be demonstrated when corrected for potential confounders, a linear mixed model was performed. The results of the linear mixed model are presented in Table 3. No direct association was observed between AIC score and post-transplant eGFR (β 0.02 (standard deviation (SD) 0.18), *p* = 0.919). Between day 50 till day 100 after transplant, eGFR increased significantly with 30.90 mL/min/1.73 m^2^ (SD 3.59, *p* < 0.001). After 100 days follow-up time, eGFR increased significantly with 11.75 mL/min/1.73 m^2^ (SD 1.80, *p* < 0.001). Patients who received a DCD graft had a lower eGFR (14.41 mL/min/1.73 m^2^ lower (SD 4.918, *p* = 0.003). An increase in donor age led to a decrease in eGFR with 0.29 mL/min/1.73 m^2^ per year of age (SD 0.08, *p* < 0.001). Furthermore, the need for postoperative dialysis and acute rejection in the first year were both associated with a lower eGFR with (9.11 mL/min/1.73 m^2^ and 6.69 mL/min/1.73 m^2^, respectively (SD 3.21, *p* = 0.005 and SD 2.82, *p* = 0.019, respectively)). The interactions between follow-up time and donor type and follow-up time and delayed graft function showed no significant addition to the model (likelihood ratio test: *p* = 0.097 and 0.134, respectively). However, a significant interaction was observed between follow-up time and ipsilateral AIC score (overall likelihood ratio test *p* < 0.001). The predicted values for eGFR during the follow-up time based on the mixed model are plotted in Figure 5, stratified according to ipsilateral AIC score quartile. This shows a sharp increase in eGFR measurements in the first period (day 0–50) for all values of ipsilateral AIC score. Further in the follow-up, it is shown that there is a relation between follow-up time and ipsilaterial AIC score; higher AIC scores show lower values of eGFR over time. The calculated GVIF values showed no important multicollinearity. Our sensitivity analysis, included in the Supplementary Table S2, showed similar results as our main analysis.

**TABLE 3:** Linear mixed model for eGFR trajectory in the first year post-transplant.

	Value	Standard error	*p*-value	(GVIF^1/2df^)^2^
(Intercept)	74.82	9.06	<0.001	
Time (days)				1.85
Day 1 – day 50	4.22	2.31	0.067	
Day 50 – day 100	30.90	3.59	<0.001	
After day 100	11.75	1.80	<0.001	
Recipient ipsilateral AIC score (per 1000 units)	0.02	0.18	0.919	2.37
Recipient age (per year)	−0.23	0.12	0.064	1.61
Recipient sex				1.36
Male	Ref			
Female	3.52	2.08	0.094	
Recipient diabetes				1.19
No	Ref			
Yes	0.33	1.93	0.865	
Recipient smoking				1.30
Never	Ref			
Currently	−0.04	2.92	0.988	
Quit	−0.08	2.25	0.973	
Previous kidney transplant				2.22
No	Ref			
Yes	0.43	3.51	0.902	
Total dialysis duration (per month)	0.03	0.06	0.621	2.54
Coronary artery disease				1.37
None	Ref			
Single vessel	5.67	3.45	0.103	
Double vessel	5.87	4.58	0.203	
Triple vessel	−2.81	3.75	0.455	
Peripheral arterial disease				1.38
No	Ref			
Yes	−4.51	4.41	0.309	
Donor type				3.24
Living	Ref			
DCD	−14.41	4.918	0.003	
DBD	−7.32	5.016	0.137	
Donor age (per year)	−0.29	0.08	<0.001	1.39
Donor diabetes				1.35
No	Ref			
Yes	−6.69	6.06	0.272	
Donor last creatinine (per µmol/l)	−0.04	0.03	0.150	1.74
Pre-emptive transplant				2.12
Yes	Ref			
No	−4.15	2.60	0.114	
Cold preservation				2.56
Static cold storage	Ref			
Hypothermic machine perfusion	0.10	3.00	0.972	
Total HLA mismatch (per 1 mismatch)	−0.21	0.63	0.744	1.23
vPRA (per %)	−0.02	0.04	0.634	2.32
Cold ischemic time (per minute)	0.00	0.01	0.917	4.87
Postoperative dialysis				2.60
No	Ref			
Yes	−9.11	3.21	0.005	
≥1 rejection episode				1.85
No	Ref			
Yes	−6.69	2.82	0.019	
Interaction time and ipsilateral calcification				1.96
Day 1 – Day 50: Ipsilateral calcification score	−0.32	0.23	0.179	
Day 50 – Day 100: Ipsilateral calcification score	−0.26	0.36	0.468	
After Day 100: Ipsilateral calcification score	−0.47	0.19	0.014	

ABOi, ABO incompatible; DBD, donation after brain death; DCD, donation after circulatory death; eGFR, estimated glomerular filtration rate; HLA, human leukocyte antigen; HLAi, human leukocyte antigen incompatible; vPRA, virtual panel reactive antibodies.

### Secondary Outcomes

The secondary outcomes are noted in Table 4 and compared between patients with a high AIC score and patients with a low AIC score. No significant differences were observed for the incidence of DGF, PNF and acute rejection within the first year after transplant. Seven patients in the high AIC group received a perioperative endarterectomy with patch angioplasty compared to none in the low AIC group, which was a statistical significant difference (*p* = 0.013). One patient in the high AIC group received a preoperative percutaneous transluminal angioplasty (PTA) with stenting compared to none in the below median AIC group (*p* = 1.000). Patient survival during the follow-up was inferior in the high AIC score group (log-rank test: *p* = 0.010). No difference was observed for death-censored graft survival.

**TABLE 4:** Secondary outcomes.

Outcome	Low AIC score (≤4,241)	High AIC score (>4,241)	*p*-value
	N = 70	N = 70	
DGF, n (%)	10 (14.3)	19 (27.1)	0.094
PNF, n (%)	3 (4.3)	4 (5.7)	1.000^a^
Acute rejection <1 year, n (%)	15 (21.4)	17 (24.3)	0.841
Perioperative vascular procedure			**0.013** **^a^**
None, n (%)	70 (100)	63 (90.0)	
Endarterectomy, n (%)	0 (0)	7 (10.0)	
Preoperative vascular procedure			1.000^a^
None, n (%)	70 (100)	69 (98.6)	
PTA, n (%)	0 (0)	1 (1.4)	
1-year graft survival (death-censored), % (CI)	94.3 (89.0–99.9)	91.4 (85.0–98.2)	0.400^b^
1-year patient survival, % (CI)	100 (not estimable)	95.7 (91.1–100.0)	0.010^b^

^a^ Fisher’s exact test.

^b^ Log-rank test.

CI, confidence interval; DGF, delayed graft function; IQR, interquartile range; PNF, primary non-function.

## Discussion

This prospective cohort study found no direct association between AIC and eGFR after transplant. The results from this study are in line with earlier studies who did not find an association between AIC and post-transplant graft function (11–14, 16). However, we found a significant interaction between time and ipsilateral calcification score, indicating that patients with a higher calcification score had a lower eGFR trajectory during the follow-up time. Other studies have not identified this interaction since none of these studies analyzed eGFR as a repeated measure over time by performing a linear mixed model. A linear mixed model is the statistical method of choice when analyzing post-transplant renal function (26). The most important reason to use this analysis is because other statistical methods do not account for drop-out. When studying renal function decline, initiation of dialysis, retransplantation and death with a function graft are the most common causes of drop-out. Because these are likely based on previously observed measurements of renal function, drop-out of the study is not completely random. Patients with high calcification scores are more likely to drop-out from the study due to death with a functioning graft because of their increased mortality risk (8, 9). Excluding these patients from the analysis results in biased estimates (26).

The pathophysiology behind the interaction that we found is not completely clear but could be speculated. Firstly, if atherosclerosis causes a hemodynamically significant stenosis with arterial lumen narrowing, this could lead to inflow problems resulting in allograft dysfunction. This has been proven in the case of transplant renal artery stenosis (27). As our study used non-contrast-enhanced CT-scan, we did not observe whether the calcification was causing a significant stenosis. Furthermore, CKD itself as well as dialysis are both important risk factors for accelerated atherosclerosis (2). Previous studies have shown that the progression of atherosclerosis slows down after transplant, but does not halt (28, 29). Therefore, it is possible that calcification progressed to a hemodynamically significant stenosis after transplant. It is also possible that intimal micro-calcification itself may already induce downstream silent ischemia and cellular necrosis to the graft, causing graft dysfunction. This mechanism has been described in studies regarding coronary artery calcification, showing that coronary artery atherosclerosis can cause significant myocardial ischemia in the absence of a hemodynamically significant stenosis due to endothelial and microvascular dysfunction (30). The last hypothesis is considered more likely because none of the patients presented with other symptoms of hemodynamically significant vascular disease during the follow-up time.

This is the first study to use a linear mixed model to assess the association between ipsilateral AIC as a continuous score and post-transplant eGFR. Most other studies used AIC as a binary variable based on the presence or absence of any calcification, a categorical variable according to the severity of calcification, or a categorization of the modified Agatston score (10-12, 14-16). Even though categorization makes interpretation of results simple, it does not reflect the underlying biology where the severity of calcification can take any number between the minimum and maximum observed value. Furthermore, classification of calcification as minimal/moderate/severe is subjective and likely to result in low inter-observer agreement. This simplification also leads to a considerable loss of power with an increased risk of a type II error (31). In our study, we used the modified Agatston score as an objective measure to quantify the amount of AIC. The Agatston score has shown before to have excellent inter-observer (spearman’s ρ ≥ 0.99) and inter-scanner agreement (spearman’s ρ ≥ 0.97) (17, 18). Our previous, dual-center, retrospective study also used the modified Agatston score to investigate a relationship between AIC and post-transplant outcomes (8). This study found an independent association between the modified Agatston score and uncensored graft survival, death with a functioning graft and cardiovascular events. Therefore, the modified Agatston score is also useful to identify patients at higher mortality risk post-transplant or patients that could benefit from more stringent cardiovascular monitoring (8).

Even though we did find a significant interaction between time and AIC, the impact of the difference in eGFR trajectory on long-term graft survival was not investigated. Prior studies found that eGFR is a good surrogate marker for graft survival (32). It can thus be stated that long-term graft survival outcomes may be inferior in patients with a high ipsilateral AIC score. The present study lacks the follow-up duration to evaluate the eGFR pattern and graft survival after 1 year. Further studies are needed to examine whether this statistical significant difference would ultimately lead to a clinically relevant difference in terms of graft survival.

Traditionally, the Agatston score has been performed by using semi-automatic software, which still requires marking of the calcified coronary artery lesions by a technician. However, a recent study showed that automatic, artificial intelligence based software had excellent correlation with the commonly used semi-automatic software (33). This allows easy calculation of the modified Agatston score on a simple, non-contrast-enhanced CT-scan. It was already shown that quantification of AIC could help identify patients at higher risk of cardiovascular mortality and events (8). The current study showed that ipsilateral AIC also negatively affects graft function over time. Therefore, standardized calculation of the modified Agatston score can help identify patients at higher risk of cardiovascular mortality and graft function decline. The simplicity of the calculation of this score, with the possibility to use an automatic algorithm, makes this score applicable in clinical practice.

Our study has several strengths and limitations. One strength is that our study is prospective, limiting selection bias. Non-contrast-enhanced CT-scan is the golden standard to measure vascular calcification which limits misclassification (34). The use of the modified Agatston score as an objective, continuous score, allowed precise quantification of AIC. A limitation of the study may be the somewhat low consent rate, which is largely due to the COVID pandemic, which caused a general study stop in our hospital. However, we do not expect that this had an impact on our results. Because we used a linear mixed model including all eGFR measurements, we had great statistical power, which allowed us to build a complex model adjusting for all factors that could potentially confound the association between calcification score and eGFR. A drawback of our linear mixed model is the complexity with the inclusion of interaction terms and splines making a direct interpretation of the estimates, or the calculation of a cut-off value, not possible.

In conclusion, this prospective cohort study found no direct relationship between ipsilateral calcification score and eGFR. However, a significant interaction between ipsilateral calcification score and follow-up time was observed, meaning that a higher calcification score is associated with a lower eGFR trajectory from 100 days after transplant. The focus should be to prevent AIC progression from an early CKD stage by promoting pre-emptive transplantation.

## Data Availability

The data that support the findings of this study are available from the corresponding author upon reasonable request.

## Abbreviations

AIC, aorto-iliac calcification; CKD, chronic kidney disease; DBD, donation after brain death; DCD, donation after circulatory death; DGF, delayed graft function; eGFR, estimated glomerular filtration rate; ESRD, end-stage renal disease; HLA, human leukocyte antigen; IQR, interquartile range; KDIGO, kidney disease improving global outcomes; PNF, primary non-function; PTA, percutaneous transluminal angioplasty; PTH, parathyroid hormone; vPRA, virtual panel reactive antibodies.
