# Supplementary material for: Ipsilateral Aorto-Iliac Calcification is Not Directly Associated With eGFR After Kidney Transplantation: A Prospective Cohort Study Analyzed Using a Linear Mixed Model
Source: Transpl Int. 2023 Jan 20;36:10647. doi: 10.3389/ti.2023.10647 (PMC9901502; doi:10.3389/ti.2023.10647)
Supplement: Supplementary file 1 [file DataSheet1.docx]

**Table S1.** Univariable estimates of all variables in the model

|  | Value | Standard Error | p-value |
| --- | --- | --- | --- |
|  |  |  |  |
| Time (days)  Day 1 – day 50  Day 50 – day 100  After day 100 | 2.40  28.2  8.86 | 1.67  2.64  1.36 | 0.152  <0.001  <0.001 |
| Recipient ipsilateral AIC score (per 1000 units) | -0.64 | 0.18 | <0.001 |
| Recipient age (per year) | -0.55 | 0.15 | <0.001 |
| Recipient sex  Male  Female | Ref  5.58 | 2.77 | 0.046 |
| Recipient diabetes  No  Yes | Ref  -5.06 | 2.77 | 0.070 |
| Recipient smoking  Never  Currently  Quit | Ref  2.20  0.41 | 4.02  3.03 | 0.586  0.894 |
| Previous kidney transplant  No  Yes | Ref  -3.81 | 3.60 | 0.291 |
| Total dialysis duration (per month) | -0.13 | 0.05 | 0.014 |
| Coronary artery disease  None  Single vessel  Double vessel  Triple vessel | Ref  -2.90  -5.34  -7.85 | 4.63  5.97  4.96 | 0.532  0.373  0.116 |
| Peripheral arterial disease  No  Yes | Ref  -8.94 | 5.91 | 0.132 |
| Donor type  Living  DCD  DBD | Ref  -24.84  -15.71 | 2.37  3.10 | <0.001  <0.001 |
| Donor age (per year) | -0.43 | 0.10 | <0.001 |
| Donor diabetes  No  Yes | Ref  -13.17 | 8.22 | 0.112 |
| Donor last creatinine (per µmol/l) | -0.03 | 0.03 | 0.303 |
| Pre-emptive transplant  Yes  No | Ref  -10.74 | 2.70 | <0.001 |
| Cold preservation  Static cold storage  Hypothermic machine perfusion | Ref  -18.14 | 2.57 | <0.001 |
| Total HLA mismatch (per 1 mismatch) | -0.57 | 0.90 | 0.530 |
| vPRA (per %) | -0.06 | 0.04 | 0.166 |
| Cold ischemic time (per minute) | -0.03 | 0.00 | <0.001 |
| Postoperative dialysis  No  Yes | Ref  -25.32 | 2.44 | <0.001 |
| ≥1 rejection episode  No  Yes | Ref  -18.16 | 2.87 | <0.001 |

*ABOi, ABO incompatible; DBD, donation after brain death; DCD, donation after circulatory death; eGFR, estimated glomerular filtration rate; HLA, human leukocyte antigen; vPRA, virtual panel reactive antibodies*

**Table S2.** Sensitivity analysis with imputation of eGFR 10 ml/min/1.73 m^2^ after graft failure if eGFR was >10 ml/min/1.73 ml^2^

|  | Value | Standard Error | p-value |
| --- | --- | --- | --- |
| (Intercept) | 75.07 | 9.06 | <0.001 |
| Time (days)  Day 1 – day 50  Day 50 – day 100  After day 100 | 4.06  30.74  11.68 | 2.31  3.60  1.80 | 0.079  <0.001  <0.001 |
| Recipient ipsilateral AIC score (per 1000 units) | 0.03 | 0.18 | 0.885 |
| Recipient age (per year) | -0.23 | 0.12 | 0.063 |
| Recipient sex  Male  Female | Ref  3.59 | 2.08 | 0.088 |
| Recipient diabetes  No  Yes | Ref  0.33 | 1.93 | 0.863 |
| Recipient smoking  Never  Currently  Quit | Ref  -0.10  -0.11 | 2.92  2.25 | 0.973  0.959 |
| Previous kidney transplant  No  Yes | Ref  0.33 | 3.51 | 0.926 |
| Total dialysis duration (per month) | 0.03 | 0.06 | 0.617 |
| Coronary artery disease  None  Single vessel  Double vessel  Triple vessel | Ref  5.66  5.83  -2.94 | 3.45  4.58  3.75 | 0.103  0.205  0.435 |
| Peripheral arterial disease  No  Yes | Ref  -4.51 | 4.41 | 0.308 |
| Donor type  Living  DCD  DBD | Ref  -14.55  -7.36 | 4.76  4.88 | 0.003  0.134 |
| Donor age (per year) | -0.29 | 0.08 | <0.001 |
| Donor diabetes  No  Yes | Ref  -6.58 | 6.06 | 0.280 |
| Donor last creatinine (per µmol/l) | -0.04 | 0.03 | 0.150 |
| Pre-emptive transplant  Yes  No | Ref  -4.16 | 2.60 | 0.113 |
| Cold preservation  Static cold storage  Hypothermic machine perfusion | Ref  0.21 | 3.00 | 0.945 |
| Total HLA mismatch (per 1 mismatch) | -0.22 | 0.63 | 0.723 |
| vPRA (per %) | -0.02 | 0.04 | 0.629 |
| Cold ischemic time (per minute) | 0.00 | 0.01 | 0.910 |
| Postoperative dialysis  No  Yes | Ref  -9.21 | 3.21 | 0.005 |
| ≥1 rejection episode  No Yes | Ref  -6.78 | 2.82 | 0.018 |
| Interaction time and ipsilateral calcification  Day 1 – Day 50: Ipsilateral calcification score  Day 50 – Day 100: Ipsilateral calcification score  After Day 100 : Ipsilateral calcification score | -0.31  -0.26  -0.47 | 0.23  0.36  0.19 | 0.188  0.467  0.014 |

*ABOi, ABO incompatible; DBD, donation after brain death; DCD, donation after circulatory death; eGFR, estimated glomerular filtration rate; HLA, human leukocyte antigen; vPRA, virtual panel reactive antibodies*

**Power calculation**

We performed a power analysis based on our primary objective (eGFR one year after transplantation). We expect a mean eGFR at 1 year follow-up of 40 (±20) ml/min/1.73m2 in the none-minimal calcification group while patients with moderate-severe calcification are expected to have a mean GFR of 30 (±20) ml/min/1.73m2. This difference is considered as clinically significant. Prior studies showed that the incidence of aorto-iliac calcifications in all kidney transplant candidates is 25%. Because the incidence of aorto-iliac calcifications is higher in older patients or patients with risk factors for aorto-iliac calcification, we expect the incidence of moderate-severe calcifications to be 33%. Therefore, we used a sampling ratio of 2:1. Our power analysis (α=0.05, β=0.2) reveals a sample size of 47 patients in the moderate/severe calcification group and 94 patients in the none/minimal calcification group
